# Comprehensive analysis of six *MED15::TFE3* renal cell carcinomas including two novel solid *MED15::TFE3* renal cell carcinomas, highlighting their morphologic, immunohistochemical and molecular differences from their cystic counterparts

**DOI:** 10.1007/s00428-026-04576-1

**Published:** 2026-05-11

**Authors:** Jung Woo Kwon, Sarah Mae Lammert, Hayley Zullow, Peng Wang, Melissa Yuwono Tjota, Tatjana Antic

**Affiliations:** Department of Pathology, The University of Chicago, Chicago, IL USA

**Keywords:** *MED15::TFE3* renal cell carcinoma, *TFE3*-rearranged renal cell carcinoma, RNA sequencing, Renal cell carcinoma

## Abstract

In 2017, the first case of *TFE3*-rearranged renal cell carcinoma (RCC) with *MED15* as a novel fusion partner gene was reported. Since then, vast majority of *MED15::TFE3* RCCs were reported to have characteristic extensively cystic appearance, with the septa being lined by tumor cells with optically clear cytoplasm and inconspicuous nucleoli, morphologically mimicking multilocular cystic renal neoplasm of low malignant potential and clear cell RCC with extensive cystic changes. IHC stains are helpful in such diagnostic scenario, as *MED15::TFE3* RCCs are negative for carbonic anhydrase IX (CAIX) and are positive for melanocytic markers and TFE3. However, the current *MED15::TFE3* RCCs case series includes two solid *MED15::TFE3* RCCs, which are morphologically, immunohistochemically and molecularly different from the conventional *MED15::TFE3* RCCs that are extensively cystic. One case is purely solid with nests of tumor cells with clear cytoplasm mimicking clear cell RCC. This purely solid *MED15::TFE3* RCC is positive for TFE3, focally positive for CAIX with box-like membranous staining, and negative for Melan A. Another case is mostly solid and tubulopapillary with minor cystic components, is positive for TFE3, and negative for Melan A and CAIX. The non-cystic *MED15::TFE3* RCCs also have unique fusion transcripts that are different from those seen in the conventional *MED15::TFE3* RCCs. The phenotypic variation of *MED15::TFE3* RCCs may be due to the property of the fusion transcripts, although additional studies are required to validate this finding.

## Introduction

*TFE3*-rearranged renal cell carcinoma (RCC) is a rare subtype of RCC that is defined by fusion of the *TFE3* gene on chromosome Xp11.23 with one of many reported partner genes, with the three most common being *PRCC*, *ASPSCR1* and *SFPQ* [1]. Morphologically, *TFE3*-rearranged RCCs are classically described to have papillary architecture composed of tumor cells with variably clear cytoplasm and are frequently associated with psammoma bodies. Immunohistochemically, *TFE3*-rearranged RCCs occasionally (depending on partner gene) show melanocytic marker positivity and the strong nuclear overexpression of TFE3 immunohistochemical (IHC) stain [2]. However, TFE3-rearranged RCC can have diverse morphologic features beyond its classic morphology, and its morphological features can widely vary depending on its fusion partner gene [1–9], often creating diagnostic challenges to pathologists.

In 2017, Classe et at reported the first case of *TFE3*-rearranged RCC with *MED15* as a novel fusion partner gene, and the tumor was described to be partially cystic [10]. Since then, numerous case reports and small case series have described over 30 cases of *MED15::TFE3* RCCs [8–21]. Vast majority of *MED15::TFE3* RCC is reported to have its characteristic extensively cystic appearance, with the cystic septa being lined by tumor cells with optically clear cytoplasm and inconspicuous nucleoli, and scattered psammomatous calcifications are often present [8, 10–17, 19, 20]. Due to its extensively cystic appearance and the tumor cells with optically clear cytoplasm, *MED15::TFE3* RCCs have significant morphologic overlaps with multilocular cystic renal neoplasm of low malignant potential (MCN-LMP), clear cell renal cell carcinoma (CCRCC) with extensive cystic changes, and clear cell papillary renal cell tumor with cystic changes (CCPRCT), and they could be easily misdiagnosed if a reviewing pathologist is unaware of the differential diagnosis in the setting of extensively cystic renal neoplasm lined by optically clear cells. IHC stains are often helpful in such diagnostic scenario, as *MED15::TFE3* RCCs are negative for carbonic anhydrase IX (CAIX), are positive for melanocytic markers, and have TFE3 nuclear overexpression.

However, the current *MED15::TFE3* RCCs case series reports two novel cases of *MED15::TFE3* RCCs with none-to-minor cystic component, which may be due to their unique underlying molecular features that were not previously reported in conventional extensively cystic *MED15::TFE3* RCCs. This series aims to provide comprehensive morphologic, immunohistochemical and molecular analysis of six *MED15::TFE3* RCCs, which include four cases that were previously reported by Doytcheva et al. [14] with additional analysis and follow up, and compare the current cases with other *MED15::TFE3* RCCs that have been reported in literature to this date.

## Methods

### Case selection, clinical data collection, and review of histopathology

The retrospective search of the University of Chicago department of Pathology archives from 2005 and 2025 resulted in identification of six *MED15::TFE3* RCCs, four of which were previously published by Doytcheva et al. [14]. Clinicopathologic data was collected from electronic medical records. All the cases were reviewed by two expert genitourinary pathologists. The study was approved by the University of Chicago Institutional Review Board.

### Immunohistochemistry

Immunohistochemical (IHC) stains were performed on selected paraffin blocks using autoimmunostainers (Leica BOND-III; Leica Biosystems, Buffalo Grove, IL, USA and BenchMark XT Ventana; Roche, Tucson, AZ, USA), with appropriate controls. IHC staining was performed for CAIX (CAIX; TH22; Leica Biosystems, Richmond, VA, USA), CK7 (CK7; OV-TL 12/30; Dako, Carpinteria, CA, USA), Melan-A (A103; Dako, Carpinteria, CA), TFE3 (SC-5958; Santa Cruz, CA, USA), and CD10 (NCL-CD10—270; Leica Biosystems, Richmond, VA, USA).

### RNA sequencing

Hybrid capture targeted RNA next-generation sequencing (NGS) analysis for gene fusion was performed using the following method. The tumor-rich tissue block was selected for cutting unstained sections. Adequate specimens should contain > 10,000 cells with > 20% of tumor cells. Appropriate number of unstained formalin-fixed paraffin-embedded tissue slides were used for DNA extraction depending on the cellularity on the sections. RNA was extracted using the RNeasy FFPE Kit (Qiagen, Valencia, CA) and quantified using a Qubit fluorometric assay (Thermo Fisher Scientific, FosterCity, CA) adjusted for the percentage of fragments greater than 100 bp using the TapeStation system (Agilent). 300 ng RNA was subjected to library prep using the KAPA stranded RNA-Seq Kit with RiboErase (Kapa Biosystems), followed by quantitation using the KAPA library quantification kit (Kapa Biosystems). Pooled libraries were captured using a panel of biotinylated oligonucleotides (xGen Lockdown probes, IDT). Amplified pooled captured libraries were sequenced in rapid run mode on a NovaSeq 6000 system (Illumina) to produce 2 × 101 bp paired end sequencing reads. Sequence data were aligned to the hg19 human reference transcriptome using STAR aligner [22], and fusions were detected using a combination of in-house developed python software and STAR fusion software. (STAR-fusion: fast and accurate fusion transcript detection from RNA-Seq. software bioRxiv120295. https://doi.org/10.1101/120295).

The assay has been validated in the University of Chicago CLIA-certified Molecular Diagnostic Laboratory to report fusions involving the following genes: *NTRK1, NTRK2, NTRK3, FGFR1, FGFR2, FGFR3, ABL1, ABL2, PDGFRA, PDGFRB, CSF1R, JAK2, P2RY8, CRLF2, KMT2A(MLL), EWSR1, TFE3, TFEB, ALK, ROS1* and* RET.*

### DNA sequencing

A representative formalin-fixed, paraffin-embedded block was selected for DNA NGS in the University of Chicago Medicine OncoPlus (UCM-OncoPlus) panel, a hybrid-capture panel targeting 1005 cancer-associated genes of which 168 genes are clinically reported. DNA extraction, DNA quantification, library preparation, and NGS were performed as described [23]. Data analysis was performed using a high-performance computing system (Center for Research Informatics, University of Chicago) using an in-house developed bioinformatics pipeline. CNV Kit14 software with additional in-house intrarun normalization with comparison with a pooled cohort of clinical controls was used to calculate copy number results. The threshold for arm-level copy number changes was an ARM average FC < 0.75 called as deleted and an ARM average FC > 1.5 called as amplified. Somatic variant calls were inspected using Integrated Genomics Viewer (Broad Institute, MIT Harvard, Cambridge, MA). Cosmic and ClinVar were used as additional tools to analyze and confirm detected variants.

### Literature review

PubMed (https://pubmed.ncbi.nlm.nih.gov/) was searched for TFE3-rearranged RCCs with *MED15::TFE3* fusion that was confirmed by molecular testing techniques (i.e. fluorescence in situ hybridization (FISH), RNA NGS, etc.). Studies that did not have molecular confirmation were excluded.

## Results

### Clinical features

Clinicopathologic features of the 6 *MED15::TFE3* RCCs are summarized in Table 1. Most patients were female (5/6, 83%) and Caucasian (4/6, 67%), with an average age of 46 years (range = 30—63). Tumor size varied greatly (range = 2.1—14.5 cm, average = 5.4 cm). All patients had no lymph nodal or distant metastasis and are alive with no evidence of recurrent/persistent disease based on their most recent follow-up data available for review.

**Table 1:** Summarized key clinicopathologic features of the 6 *MED15::TFE3* RCCs in the current study

**Case #**	**Age/Sex**	**Procedure**	**Size (cm)**	**Stage**	**Architecture**	**Melan A**	**TFE3**	**CAIX**	**CK7**	**CD10**	**P504S**	**Exons Fused**	**Follow up**
1*	37/F	Partial nephrectomy	14.5	pT2bNxMx	Extensively cystic	+	+	-	-	+	+	*MED15* exon 8-*TFE3* exon 6	NED 67 months
2*	52/F	Partial nephrectomy	1.8	pT1aNxMx	Extensively cystic	+	+	-	-	+	+	*MED15* exon 9-*TFE3* exon 6	NED 62 months
3*	35/F	Partial nephrectomy	4.5	pT1NxMx	Extensively cystic	+	+	-	-	+	+	*MED15* exon 8-*TFE3* exon 6	NED 11 months
4*	63/F	Partial nephrectomy	2.1	pT1NxMx	Extensively cystic	+	+	-	-	+	+	*MED15* exon 9-*TFE3* exon 6	NED 8 months
5	30/M	Radical nephrectomy	3.1	pT3aNxMx	Entirely solid	-	+	+ (focal)	-	+	+	*MED15* exon 11-*TFE3* exon 7; *MED15* mid-intron 11-*TFE3* exon 6	NED 5 months
6	61/F	Radical nephrectomy	6.5	pT3aN0Mx	Predominantly tubulopapillary with minor cystic component	-	+	-	-	+	+	*MED15* exon 13-*TFE3* exon 4	NED 134 months

F = Female, M = Male

NED = No evidence of disease

^*^Previously published by Doytcheva et al.[14]

### Cases 1—4: Conventionally described extensively cystic MED15::TFE3 RCCs

**Fig. 1:**
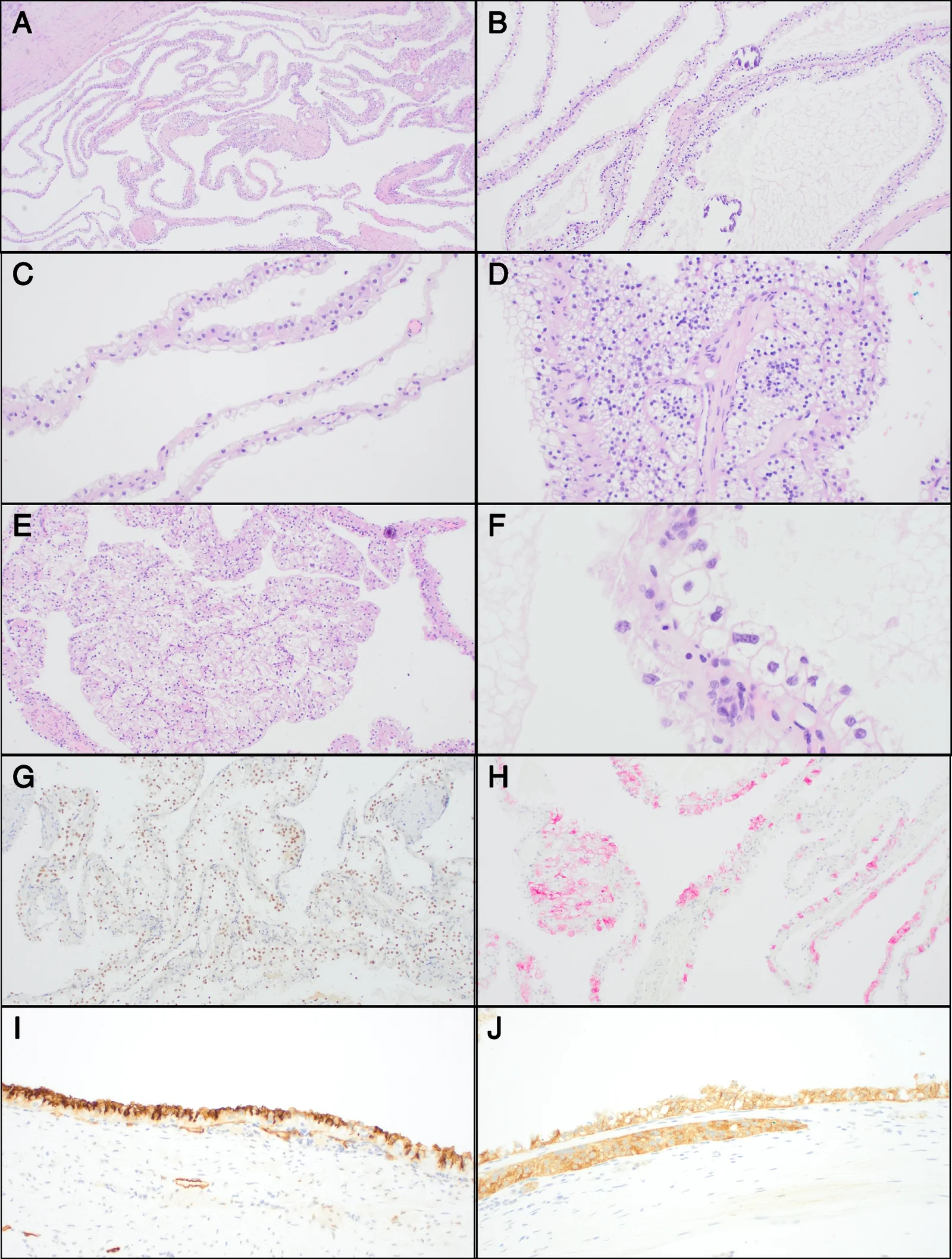
Cases 1—4 showed extensive cystic architecture (**A**). The cystic wall had thin fibrous septa with scattered psammomatous calcifications (**B**). The cystic wall was lined by tumor cells with optically clear cytoplasm and small nuclei with inconspicuous nucleoli (**C**). Focal microscopic areas showed solid (**D**) and papillary-appearing proliferation (**E**), although the tumor cells seen in this area had identical cytologic features to those lining the cystic wall. In some areas, multinucleated tumor cells were identified, although the nuclear features were identical to the adjacent mononuclear tumor cells (**F**). IHC stains showed that all the cases were diffusely positive for TFE3 (**G**), Melan A (**H**), CD10 (apical pattern;** I**), P504S (**J**) and PAX8, and negative for CK7 and CAIX

Cases 1—4, previously published by Doytcheva et al. [16], grossly revealed well-circumscribed extensively multiloculated cystic lesion without solid areas. H&E showed extensive cystic architecture with no gross solid areas, which corresponded to their gross appearances (Fig. 1A). The cystic wall had thin fibrous septa with scattered psammomatous calcifications (Fig. 1B). The cystic wall was lined by tumor cells with optically clear cytoplasm and small nuclei with inconspicuous nucleoli (Fig. 1C). Focal microscopic areas showed solid (Fig. 1D) and papillary-appearing proliferation (Fig. 1E), although the tumor cells seen in this area had identical cytologic features to those lining the cystic wall. In some areas, multinucleated tumor cells were identified, although the nuclear features were identical to the adjacent mononuclear tumor cells (Fig. 1F). Tumor necrosis and mitotic figures were not identified. IHC stains with appropriate controls showed that Cases 1—4 were diffusely positive for TFE3 (Fig. 1G), Melan A (Fig. 1H) and PAX8, and negative for CK7 and CAIX.

RNA sequencing of Cases 1—4 confirmed the presence of *MED15::TFE3* fusion transcripts in all cases. The fusion transcript in Cases 1 and 3 showed a connection between exon 8 of *MED15* and exon 6 of *TFE3*. The fusion transcript in Cases 2 and 4 showed a connection between exon 9 of *MED15* and exon 6 of *TFE3*. All fusion transcripts kept the reading frames preserved at the junction points.

### Case 5: Novel case of purely solid MED15::TFE3 RCC morphologically mimicking clear cell renal cell carcinoma

**Fig. 2:**
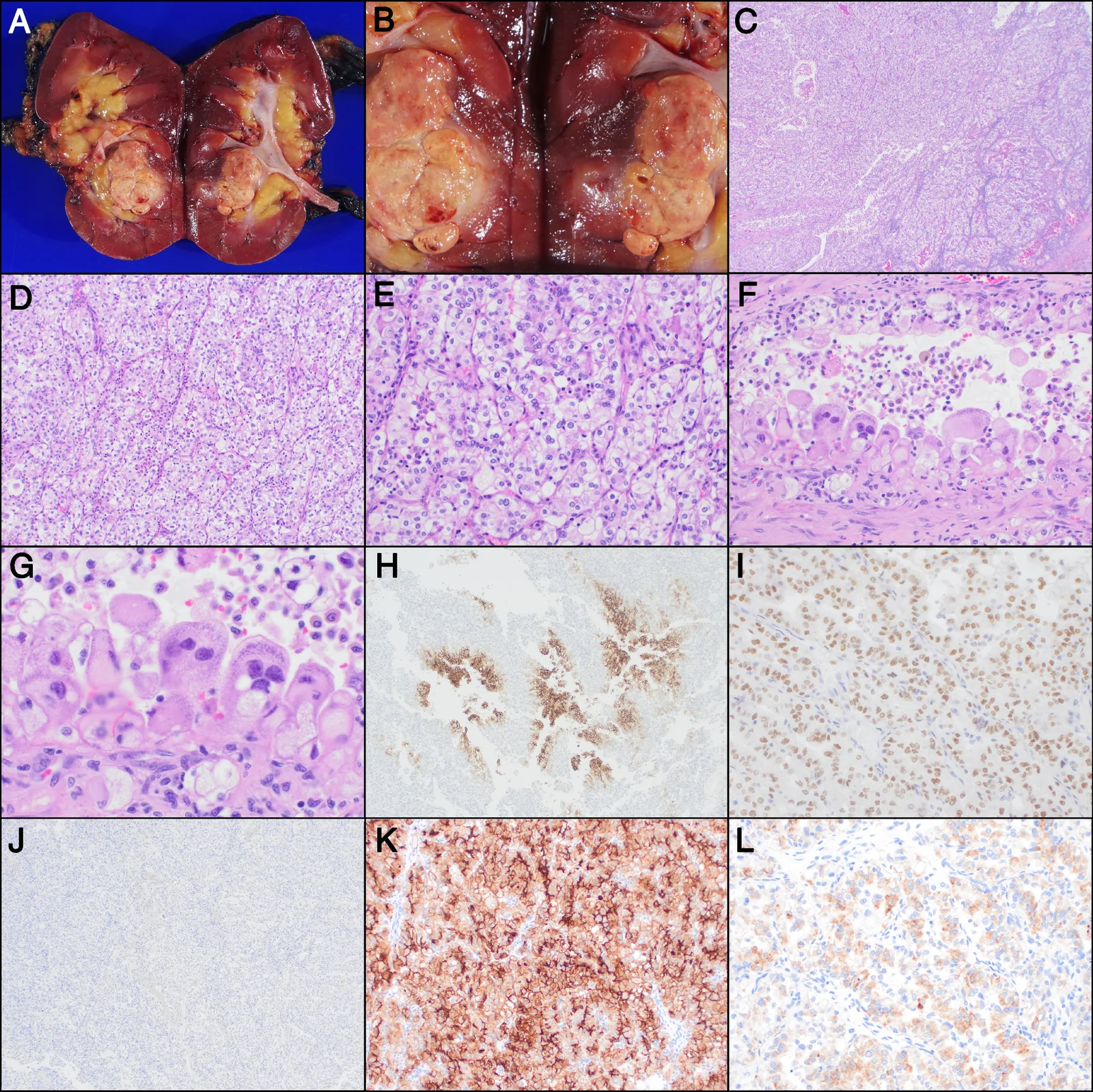
Case 5 grossly revealed purely solid yellow-beige multinodular mass with no cystic areas (**A-B**). H&E revealed a tumor with purely solid nested architecture with areas of hemorrhage (**C**). The tumor nests were predominantly composed of tumor cells with cleared-out cytoplasm, surrounded by rich thin-walled capillary network (**D**), highly reminiscent of CCRCC. Tumor cells had uniformly round small-to-medium sized nuclei with inconspicuous nucleoli, and some displayed variable degree of eosinophilia (**E**). There were also rare foci with degenerative changes, where there were “balloon” cells with ample eosinophilic cytoplasm (**F**). In these degenerative foci, bizarre eosinophilic multinucleated giant tumor cells with variable intracytoplasmic vacuolation and blue granules/basophilic stippling were also present (**G**). IHC stains showed that the tumor had focal (5—10%) box-like membranous staining for CAIX (**H**), primarily centered around the foci of degeneration/fragmentation. TFE3 IHC stain showed nuclear overexpression (**I**). Tumor was negative for Melan A (**J**) and CK7. Additionally, tumor was diffusely positive for CD10 (**K**) and also showed patchy positivity for P504S (**L**)

Case 5 grossly revealed purely solid yellow-beige multinodular mass with no cystic areas (Fig. 2A-B). H&E revealed a tumor with purely solid nested architecture with areas of hemorrhage (Fig. 2C). No cystic component was seen. The tumor nests were predominantly composed of tumor cells with cleared-out cytoplasm, surrounded by rich thin-walled capillary network (Fig. 2D), highly reminiscent of CCRCC. Tumor cells had uniformly round small-to-medium sized nuclei with inconspicuous nucleoli, and some displayed variable degree of eosinophilia (Fig. 2E). There were also rare foci with degenerative changes, where there were “balloon” cells with ample eosinophilic cytoplasm (Fig. 2F). In these degenerative foci, bizarre eosinophilic multinucleated giant tumor cells with variable intracytoplasmic vacuolation and blue granules/basophilic stippling were also present (Fig. 2G). Tumor necrosis, increased mitotic activity and psammomatous calcifications were not identified.

IHC stains with appropriate controls showed that the tumor had focal (5—10%) box-like membranous staining for CAIX (Fig. 2H), primarily centered around the foci of degeneration/fragmentation. TFE3 IHC stain showed nuclear overexpression (Fig. 2I). Tumor was negative for Melan A (Fig. 2J) and CK7. Additionally, tumor was diffusely positive for CD10 (Fig. 2K) and also showed patchy positivity for P504S (Fig. 2L).

RNA sequencing of Case 5 showed two distinct *MED15::TFE3* fusion transcripts. One fusion transcript showed a connection between exon 11 of *MED15* and exon 7 of *TFE3*, and another showed a connection between mid-intron 11 of *MED15* and middle of exon 6 of *TFE3*. Both transcripts kept the reading frames preserved at the junction points.

DNA sequencing showed no pathogenic mutations in *VHL* or other RCC-defining genes (i.e. *TSC1*, *TSC2*, *MTOR*). No significant copy number changes were identified. There was no microsatellite instability.

### Case 6: Predominantly tubulopapillary MED15::TFE3 RCC with minor cystic components

**Fig. 3:**
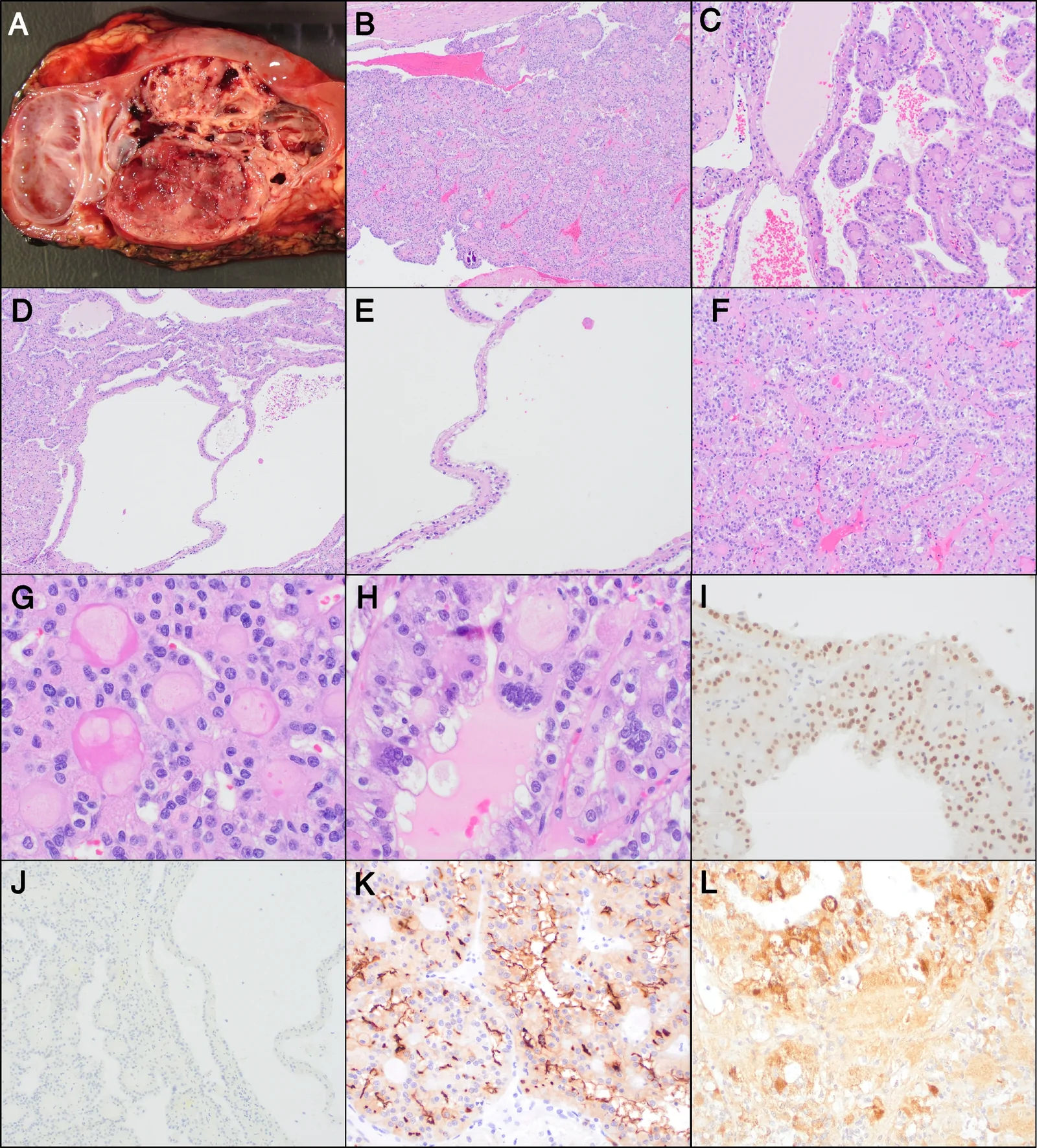
Case 6 grossly revealed a fleshy solid mass with areas of cystic changes that focally showed excrescences and polypoid changes (**A**). H&E revealed tumor cells forming a tightly packed tubulopapillary architecture forming a solid configuration with scattered psammomatous calcification (**B**) and minor cystic components (**C** and **D**). The minor cystic component showed thin fibrous septa lined by tumor cells with clear cytoplasm with occasional eosinophilic granularity (**E**), highly reminiscent of the morphologic features seen in Cases 1—4. In areas with tubulopapillary architecture, tumor cells were predominantly eosinophilic with occasional clear cytoplasm (**F**). Tumor cells had round small-to-medium sized nuclei with inconspicuous nucleoli, and it occasionally showed amorphous material in the lumen that was reminiscent of corpora amylacea in prostatic lumina (**G**). In some areas, there were multinucleated giant tumor cells with syncytial appearance, although their nuclei seemed similar to the adjacent mononuclear tumor cells (**H**). IHC stains showed that the tumor was positive for TFE3 (**I**), and negative for Melan A (**J**), CAIX and CK7. Additionally, tumor was positive for CD10 (apical pattern; **K**) and also showed patchy positivity for P504S (**L**)

Case 6 grossly revealed a fleshy solid mass with focal cystic changes that showed excrescences and polypoid architecture. (Fig. 3A). H&E revealed tumor cells forming a tightly packed tubulopapillary architecture forming a solid configuration with scattered psammomatous calcification (Fig. 3B) and minor cystic components (Figs. 3C and 3D). The minor cystic component showed thin fibrous septa lined by tumor cells with clear cytoplasm with occasional eosinophilic granularity (Fig. 3E), highly reminiscent of the morphologic features seen in Cases 1—4. In areas with tubulopapillary architecture, tumor cells were predominantly eosinophilic with occasional clear cytoplasm (Fig. 3F). Tumor cells had round small-to-medium sized nuclei with inconspicuous nucleoli, and it occasionally showed amorphous material in the lumen that was reminiscent of corpora amylacea in prostatic lumina (Fig. 3G). In some areas, there were multinucleated giant tumor cells with syncytial appearance, although their nuclei seemed similar to the adjacent mononuclear tumor cells (Fig. 3H). Tumor necrosis and mitotic figures were not identified.

IHC stains with appropriate controls showed that the tumor was positive for TFE3 (Fig. 3I), and negative for Melan A (Fig. 3J), CAIX and CK7. Additionally, tumor was positive for CD10 (apical pattern; Fig. 3K) and also showed patchy positivity for P504S (Fig. 3L).

RNA sequencing of Case 6 confirmed the presence of a *MED15::TFE3* fusion transcript, which showed a connection between exon 13 of *MED15* and exon 4 of *TFE3*. The fusion transcript kept the reading frames preserved at the junction points.

DNA sequencing showed no pathogenic mutations in *VHL* or other RCC-defining genes (i.e. *TSC1*, *TSC2*, *MTOR*). No significant copy number changes were identified. There was no microsatellite instability.

## Discussion

Since Classe et al. first reported a case of *TFE3*-rearranged RCC with *MED15* as a novel fusion partner gene in 2017 [10], a total of 33 cases (excluding the 6 cases in the current study) of molecularly confirmed *MED15::TFE3* RCC has been reported in literature to this date [8–21], and their key features are summarized in Table 2. Among the case reports/series that reported architectural patterns of their tumors (excluding a total of 4 cases by Pei et al. [9], Harada et al. [17] and Achom et al. [21]), all 29 cases have been described to have a significant amount of cystic component. In fact, 86% (25 of 29 cases) of *MED15::TFE3* RCCs have been described to be extensively or exclusively cystic. The remaining 14% (4 of 29 cases) of *MED15::TFE3* RCCs were described to be partially cystic with other mixed architectural components, such as solid, papillary, tubular and acinar patterns [8, 10, 12]. Interestingly, all reported cases of *MED15::TFE3* RCCs that were stained for Melan A immunostain showed at least some positivity (12/12), although some cases that were stained for HMB45 and MART-1 immunostains showed negativity.

**Table 2:** Summarized key clinicopathologic features of all the reported *MED15::TFE3* RCCs in the literature

Study	Age/Sex	Size (cm)	Architecture	Exons fused	Outcome	Melanocytic marker
Classe et al. [10]	34/F	9.5	Mixed and cystic	*MED15* exon 9-*TFE3* exon 6	NED 48 months	N/A
Wang et al. [8]	42/M	1.5	Extensively cystic	N/A	NED	Melan-A +
	41/M	9	Acinar, tubular & papillary*	N/A	Alive; Lung metastases after 15 years	Melan-A +
	54/F	4	Extensively cystic	N/A	NED	Melan-A +
	45/F	4.5	Extensively cystic	N/A	NED	Melan-A +
	30/F	5	Cystic, papillary, solid	N/A	NED	Melan-A +
Ye et al. [12]	35/F	5	Solid and cystic	*MED15* exon 11-*TFE3* exon 6	N/A	Neg (Melan-A & HMB45)
Pei et al. [9]	22/F	2	N/A	N/A	N/A	Melan-A 25% +, HMB45 -
Song et al. [13]	40/M	4	Extensively cystic	N/A	NED 46 months	Mart-1 +
	51/F	3.6	Extensively cystic	N/A	NED 38 months	Negative (Mart-1)
	49/M	3	Extensively cystic	N/A	NED 54 months	Negative (Mart-1)
	47/M	5.8	Extensively cystic	N/A	NED 8 months	Negative (Mart-1)
	28/F	3	Extensively cystic	N/A	NED 19 months	Mart-1 +
	32/F	3.1	Extensively cystic	N/A	NED 56 months	Mart-1 +
Harada et al. [17]	N/A	N/A	N/A	N/A	N/A	N/A
Argani et al. [11]	74/F	6	Extensively cystic	N/A	N/A	Melan-A +, HMB45 -
	40/F	3	Extensively cystic	N/A	N/A	Melan-A +, HMB45 -
Cai et al. [19]	71/F	3.8	Cystic, multiloculated	*MED15* exon 9-*TFE3* exon 6	NED 33 months	Melan-A +
Sun et al. (8 cases) [20]	Median age (range): 37.5 (18—70); 6 females, 2 males					
	TFE3—06	5.9	Extensively cystic	N/A	NED	N/A
	TFE3—21	4.1	Extensively cystic	N/A	NED	N/A
	TFE3—27	4.5	Extensively cystic	N/A	NED	N/A
	TFE3—31	3.3	Extensively cystic	N/A	NED	N/A
	TFE3—38	13.4	Extensively cystic	N/A	NED	N/A
	TFE3—51	5.8	Extensively cystic	N/A	NED	N/A
	TFE3—60	6.5	Extensively cystic	N/A	NED	N/A
	TFE3—63	6	Extensively cystic	N/A	NED	N/A
Yin et al. [14]	29/F	9.3	Extensively cystic	*MED15* exon 8-*TFE3* exon 6	NED 26 months	Melan-A +, HMB45 -
	35/F	4.8	Extensively cystic	*MED15* exon 7-*TFE3* exon 6	NED 6 months	Melan-A +, HMB45 -
Mladenovic et al. [15]	36/F	4.7	Exclusively cystic	N/A	NED 13 months	Melan-A + (patchy)
Deng et al. [16]	27/F	6.8	Cystic	N/A	NED 71 months	N/A
	17/F	16	Cystic	N/A	NED 47 months	N/A
Achom et al. [21]	TRCC_10 (age N/A; female)	N/A	N/A	N/A	N/A	N/A
	TRCC_13 (age N/A; female)	N/A	N/A	N/A	N/A	N/A
**Current study**	37/F** **(Case 1)**	**14.5**	**Extensively cystic**	***MED15* exon 8-*TFE3* exon 6**	**NED 67 months**	**Melan-A + **
	52/F** **(Case 2)**	**1.8**	**Extensively cystic**	***MED15* exon 9-*TFE3* exon 6**	**NED 62 months**	**Melan-A + **
	35/F** **(Case 3)**	**4.5**	**Extensively cystic**	***MED15* exon 8-*TFE3* exon 6**	**NED 11 months**	**Melan-A + **
	63/F** **(Case 4)**	**2.1**	**Extensively cystic**	***MED15* exon 9-*TFE3* exon 6**	**NED 8 months**	**Melan-A + **
	**30/M** **(Case 5)**	**3.1**	**Entirely solid**	***MED15* exon 11-*TFE3* exon 7;** ***MED15* mid-intron 11-*TFE3* exon 6**	**NED 5 months**	**Negative (Melan A)**
	**61/F** **(Case 6)**	**6.5**	**Solid & papillary with minimal cystic component**	***MED15* exon 13-*TFE3* exon 4**	**NED 134 months**	**Negative (Melan A)**

^*^This is the morphology of the primary kidney tumor (case 19) described in the main text by Wang et al. [8]. Lung metastasis of the tumor that developed later in the same patient had solid architecture

^**^Previously published by Doytcheva et al.[14]

In the current study, four of the six cases showed conventional features of *MED15::TFE3* RCC that was previously reported: extensively cystic appearance morphologically mimicking MCN-LMP, CCRCC with extensive cystic changes, and clear cell papillary renal cell tumor (CCPRCT) with cystic appearance. As they have significant morphologic overlap, one’s threshold for ordering IHC stains should be low. A panel of CAIX, CK7, melanocytic markers such as HMB45, Melan A, and MART-1, and TFE3 could be useful, as cystic *MED15::TFE3* RCCs are negative for CAIX (which is positive in MCN-LMP, CCRCC, and CCPRCT) and CK7 (which is always positive in CCPRCT and also variably positive in MCN-LMP), are positive for melanocytic markers (which are negative in MCN-LMP, CCRCC, and CCPRCT), and have TFE3 nuclear overexpression. In particular, CAIX negativity should raise suspicion for *MED15::TFE3* RCC in cystic lesions with clear cells, as MCN-LMP, CCRCC, and CCPRCT, the main differential diagnosis in this diagnostic scenario, are all diffusely positive for CAIX.

However, the remaining two *MED15::TFE3* RCCs had none-to-minimal cystic components, with one case being the first novel reported case of purely solid *MED15::TFE3* RCC. The purely solid case (Case 5) of *MED15::TFE3* RCC in the current study showed striking morphologic mimicry of CCRCC, as it is mostly composed of solid nests of tumor cells with clear cytoplasm that are surrounded by thin-walled capillaries. It had unusual degenerative areas that showed multinucleated giant tumor cells and bizarre “balloon” cells, although the multinucleated giant cells and enlarged eosinophilic tumor cells can be seen in clear cell renal cell carcinoma, with the latter more frequently seen in high grade clear cell renal cell carcinomas, particularly those with *BAP1* mutation [24–31]. Additionally, this purely solid *MED15::TFE3* RCC did not have any psammomatous calcifications, which was present in all other *MED15::TFE3* RCCs in the current study. However, the presence of the psammomatous calcifications would not necessarily distinguish this tumor from CCRCCs as some CCRCCs are reported to have psammomatous calcifications [28]. Presence of the blue granules/basophilic stippling in this purely solid *MED15::TFE3* RCC is however unreported in CCRCC and is a feature that was thought to be primarily linked to eosinophilic solid and cystic RCC (ESC RCC) [32–34]. However, the presence of the blue granules/basophilic stippling may not be a specific morphologic feature as they have been described to be present in other TSC/MTOR/folliculin/TFE3/TFEB pathway mutated renal tumors apart from ESC RCC [35, 36].

While the morphologic features of this purely solid *MED15::TFE3* RCC are highly similar to those of CCRCC, its IHC profile could be helpful in distinguishing it from CCRCC, although some caveats exist. Similar to the conventional *MED15::TFE3* RCCs that are extensively cystic, the purely solid *MED15::TFE3* RCC was positive for TFE3, and mostly negative for CAIX, which would be highly unusual for CCRCC. However, it did show focal strong box-like membranous positivity for CAIX, primarily in the areas of fragmentation/degeneration, and one should be mindful to refrain from misinterpreting this focal membranous positivity as evidence supporting the diagnosis of CCRCC as CCRCC usually has diffuse box-like membranous staining, not focal. Additionally, as the CD10 positivity was seen in this purely solid *MED15::TFE3* RCC, as it is often seen in CCRCC, CD10 positivity should be interpreted with caution, especially in the setting where diffuse membranous CAIX positivity is not seen. Interestingly, this purely solid *MED15::TFE3* RCC was negative for Melan-A, and one should be mindful that *TFE3*-rearranged RCCs can be negative for melanocytic markers and should not hastily exclude *TFE3*-rearranged RCC based on the melanocytic marker negativity. Conversely, TFE3 positivity, while it is a helpful clue towards the diagnosis of *TFE3*-rearrnaged RCC, can have false positives in various renal tumors such as *FH*-deficient RCC [37], *ALK*-rearranged RCC [38], and *FLCN*-mutant renal epithelial tumors [39], and should be interpreted with caution. In challenging cases where the distinction between CCRCC and purely solid *MED15::TFE3* RCC is difficult, molecular tests could aid in definitive classification, as CCRCC has its characteristic *VHL* mutation and/or loss of chromosome 3p and no *TFE3* rearrangement, and vice versa for the *MED15::TFE3* RCC. As CCRCC and the current case of purely solid *MED15::TFE3* RCC have significant morphologic overlap, one’s threshold for ordering ancillary IHC and/or molecular tests should be low, especially when it can lead to different clinical managements. Basic IHC panel of CAIX and TFE3 could be very useful in this setting, and NGS and/or *TFE3* fluorescent in situ hybridization (FISH) could also aid in definitive classification if necessary.

Case 6 in the current study also showed an unconventional morphology for a *MED15::TFE3* RCC as it was predominantly solid and tubulopapillary with only minor cystic components. Morphologically, it had some features that are reminiscent of (formerly) papillary RCC (PRCC), type 1, although the presence of psammomatous calcifications and the tumor cells with clear cytoplasm, especially in the minor cystic component, should alert one to promptly order additional work up. TFE3 and CK7 can be helpful in this diagnostic scenario, as CK7 is negative in *MED15::TFE3* RCC and usually positive in (formerly) PRCC, type 1, and TFE3 is positive in *MED15::TFE3* RCC and negative in (formerly) PRCC, type 1, although the same caveat exists for the interpretation of TFE3 as described above. It must be noted that CD10 and P504S should be interpreted with caution in this scenario, as Case 6 showed apical CD10 staining pattern and P504S positivity, similar to the patterns observed in PRCC. Interestingly, Case 6 was negative for Melan A in both cystic and tubulopapillary components similar to the purely solid *MED15::TFE3* RCC (Case 5), similar to Case 5.

To this date, there is no report of patients with *MED15::TFE3* RCC dying from the disease. There was only one reported case of metastasis, which was from the cohort by Wang et al. [8], and the patient is still alive after 15 years. Data to this date suggest that *MED15::TFE3* RCCs are mostly indolent-behaving compared to the most *TFE3*-rearranged RCCs, the prognosis of which could vary depending on the fusion partner gene [1, 6, 40, 41]. Given the indolent nature of the tumor, including the ones with solid architecture, distinguishing them from CCRCC, which has more aggressive behavior, could be clinically important as the accurate distinction may affect subsequent clinical follow up and work up.

Additionally, some interesting molecular-phenotypic correlations were observed between the conventional extensively cystic *MED15::TFE3* RCCs and those with unusual morphologic features (Cases 5—6). Conventional *MED15::TFE3* RCCs that are extensively cystic with Melan-A positivity uniformly have fusion transcripts that show a connection to exon 6 of *TFE3*, although the connection point of *MED15* can vary. However, the non-cystic *MED15::TFE3* RCCs with Melan-A negativity (Cases 5—6) both have fusion transcripts that show a connection to different exons other than the exon 6, with Case 5 with a transcript showing a connection to exon 7 of *TFE3* and Case 6 with a transcript showing a connection to exon 4 of *TFE3*. Additionally, the purely solid *MED15::TFE3* RCC (Case 5) is unique compared to all other *MED15-TFE3* RCCs as it has two distinct *MED15::TFE3* fusion transcripts with one of them having an intron of *MED15* as a connection point. This is the only reported case of *MED15::TFE3* RCCs that has two distinct fusion transcripts and have an intron as a connection point and also happens to be the only reported case of purely solid *MED15::TFE3* RCCs. Although it is unclear what the functional impact of these various fusion transcripts is, these findings imply that the connection points of the fusion transcripts may have impact on tumor phenotypes even within the *TFE3* fusion with the same partner gene of *MED15*. Although this is an extrapolation, these findings could also imply that the property of the fusion transcripts itself may contribute to the heterogeneity of tumor phenotypes observed in tumors with specific driver pathogenic fusion transcripts with the same partner gene. However, further studies with additional cases are required to validate this finding.

To conclude, this study reports two solid *MED15::TFE3* RCCs with morphologic, immunohistochemical, and molecular features that are different from their cystic counterpart. In particular, the purely solid *MED15::TFE3* RCC in this case morphologically mimic CCRCC and it would be important for pathologists to recognize when to order ancillary studies to confirm or exclude the diagnosis as the accurate distinction may have clinical consequences. Phenotypic variation within the *MED15::TFE3* RCCs may be due to the innate property of the fusion transcripts, although additional studies are required to validate this finding.
